# Assessing the translational relevance of specific molecular pathways in spontaneous lupus mouse models

**DOI:** 10.3389/fimmu.2026.1823650

**Published:** 2026-05-13

**Authors:** María Rivas-Torrubia, María Morell, Zuzanna Makowska, Jorge Kageyama, Anne Bosshard, Julius Lindblom, Maria Orietta Borghi, Eleonore Bettacchioli, PRECISESADS flow cytometry consortium, PRECISESADS clinical consortium, Ioannis Parodis, Lorenzo Beretta, Concepción Marañón, Jacques-Olivier Pers, Ralf Lesche, Fiona McDonald, Marta E. Alarcón-Riquelme, Guillermo Barturen

**Affiliations:** 1 Genyo. Pfizer–University of Granada–Junta de Andalucía Centre for Genomics and Oncological Research, Granada, Spain; 2 Bayer Pharma AG, Berlin, Berlin, Germany; 3 Division of Rheumatology, Department of Medicine Solna, Karolinska Institutet and Karolinska University Hospital, Stockholm, Sweden; 4 Immunorheumatology Research Laboratory, IRCCS Istituto Auxologico Italiano, Milan, Italy; 5 Department of Clinical Sciences and Community Health, University of Milan, Milan, Italy; 6 Lymphocytes B, Autoimmunité et Immunothérapies (LBAI), UMR 1227, Institut National de la Santé et de la Recherche Médicale (INSERM), University of Western Brittany, Brest, France; 7 Department of Immunology and Immunotherapy Laboratory, University-Hospital of Brest, Brest, France; 8 Department of Rheumatology, Faculty of Medicine and Health, Örebro University, Örebro, Sweden; 9 Referral Center for Systemic Autoimmune Diseases, Fondazione IRCCS Ca’ Granda Ospedale Maggiore Policlinico di Milano, Milano, Italy; 10 Institute for Environmental Medicine, Karolinska Institute, Stockholm, Sweden; 11 Department of Genetics, Faculty of Science, University of Granada, Granada, Spain; 12 Bioinformatics Laboratory, Biotechnology Institute, Centro de Investigación Biomédica, Parque Tecnológico de Ciencias de la Salud (PTS), Granada, Spain

**Keywords:** autoantibodies, cytokines, flow cytometry, molecular integration, mouse models, systemic lupus erythematosus

## Abstract

**Background:**

Systemic lupus erythematosus (SLE) is a complex autoimmune disease characterized by loss of self-tolerance, causing inflammation and tissue damage in multiple organs. Although animal models have advanced our understanding of SLE’s molecular basis, recent regulatory changes and longstanding concerns regarding reproducibility and translatability have renewed the need to critically evaluate how these models mirror human disease. Understanding pathway-level similarities and differences between mouse models and human disease is essential, given the marked clinical and molecular heterogeneity of SLE.

**Methods:**

Four spontaneous SLE mouse models were studied: MRL*^lpr/lpr^*, NZB/W, BXSB.*Yaa*, and Tlr7.Tg6. Transcriptome sequencing from blood, spleen, and kidney; flow cytometry from the spleen; and cytokines and autoantibody measurement in plasma were performed at four time points. Similar molecular datasets from the human PRECISESADS SLE cohort were used for the integration.

**Results:**

The study identified specific molecular pathways driving the phenotype in each mouse model and established a framework describing the dynamics of these phenotype-associated molecular signatures, thereby facilitating the selection of time points of interest for future mouse-oriented experimental designs. In addition, by comparing these pathways with those observed in human SLE, we identified the most similar ones and their relationship with disease activity, providing crucial insight into their translational relevance. Importantly, disease severity across models was linked to both the extent and timing of molecular dysregulations. As expected, MRL*^lpr/lpr^* showed the most aggressive phenotype with early immune activation and apoptosis dysregulation, while Tlr7.Tg6 presented late-onset signatures associated with interferon and inflammation. Shared molecular features with human SLE included interferon responses, T and B cell depletion, and neutrophil activation. Integration analysis revealed distinct, yet overlapping, immune pathways between models and species, with some signatures such as age-associated B cells and double-negative memory T cells being model-specific but potentially relevant to early disease processes.

**Conclusions:**

These findings provide a valuable framework for future SLE research and reinforce the utility of mouse models for studying specific molecular pathways related to human SLE pathogenesis and heterogeneity. The integration of longitudinal mouse and human molecular information highlights the models that best recapitulate key aspects of human disease, offering guidance for the study of specific immunopathological mechanisms or therapeutic targets.

## 1 Introduction

The mouse has been a key model organism for studying human diseases and has aided our understanding of the molecular dysregulation underlying human phenotypes. However, concerns about the reproducibility and translatability of mouse model findings to humans, particularly in preclinical trials, have grown in recent years. The recent authorization of the U.S. Food and Drug Administration (FDA) to proceed with human trials without requiring prior drug safety and efficacy testing in animals (1), has intensified discussions about the role and relevance of animal models in modern biomedical research, especially for complex, heterogeneous diseases.

Animal modeling of complex diseases, such as systemic lupus erythematosus (SLE), is particularly challenging because of their clinical and molecular heterogeneity (2–4). Recent publications have shown that different subgroups of clinically diagnosed SLE patients show different molecular pathways dysregulations during the course of the disease (5, 6), some of which are shared with other systemic autoimmune diseases (7). This molecular heterogeneity may partly explain the failure of some randomized clinical trials evaluating treatments commonly used for other diseases, or even for SLE in the clinical practice (8).

Numerous mouse models exist for studying SLE, although none fully replicates the entire disease’s clinical spectrum. Each model exhibits human-like phenotypes and offers specific characteristics of preclinical interest (9, 10), mimicking the molecular heterogeneity found in SLE patients. Broadly, lupus models are categorized as induced, useful for studying environmental triggers, or spontaneous, which arise from genetic mutations or gene duplications and allow investigation of inherited susceptibility and mechanistic pathways. Therefore, selecting the appropriate model and time points is essential for dissecting SLE−related biological processes and for designing informative preclinical studies (11–13).

In this work, we studied four spontaneous mouse models: BXSB.*Yaa*, Tlr7.Tg6, MRL*^lpr/lpr^*, and NZB/W. Both BXSB.*Yaa* and Tlr7.Tg6 involve *Tlr7* gene duplication; in BXSB.*Yaa* the entire Y-autoimmune accelerator locus is translocated in males from the X to the Y chromosome. The *Tlr7* gene and the *Yaa* locus regulate the activation of the type I interferon pathway by ribonucleic acid complexes, a critical pathway in the pathogenesis of SLE (14). Despite sharing *Tlr7* gene duplication, these models differ in cell-population expansion, inflammatory pathways, autoantibody positivity, and disease progression (15, 16). The MRL*^lpr/lpr^* model, driven by the *lpr* mutation in the *Fas* gene, leads to defective immune-cell apoptosis, resulting in the accumulation of CD4-CD8 double-negative T cells and the induction of the disease due to the amplification of susceptibility genes. It is a model with rapid and severe development compared to other spontaneous models (16). Lastly, the NZB/W model is an F1 hybrid between the New Zealand Black (NZB) and the New Zealand White (NZW) strains. It is characterized by early elevated antinuclear antibodies (ANAs) levels and other diverse autoantibodies which lead to immune complex-mediated nephritis (16).

This study profiled similar molecular data from different omic layers in mouse and human patients from the PRECISESADS SLE cohort (7). Given the heterogeneity of SLE and the diversity of existing mouse models, we reasoned that integrating patient and model data across multiple molecular layers and tissues was crucial for identifying shared molecular features between human disease and mouse phenotypes. Using molecular data integration factorization methodology, we aimed to establish a framework for the future design of preclinical studies in SLE, highlighting the models that exhibit molecular signatures similar to those associated with human disease activity and the time required for the models to develop human-like molecular signatures.

## 2 Methods

### 2.1 Cohorts and study design

#### 2.1.1 Human cohort

Whole blood samples from 342 SLE patients and 497 healthy controls were obtained from the PRECISESADS cohort (**Supplementary Table 1**). Recruitment criteria and detailed information on sampling and molecular profiling can be found in Barturen et al. (7).

#### 2.1.2 Mouse models

Whole blood, spleen, and kidney were sampled at four time points for four SLE spontaneous mouse models (MRL*^lpr/lpr^*, NZB/W F1, BXSB.*Yaa*, and Tlr7.Tg6), as well as for their respective genetic controls (MRL/J, NZW, BXSB, C57BL/6). Because the severity and the kinetics of the SLE development differ among models, we used different times to better align phenotype progression across models. In MRL*^lpr/lpr^*, samples were taken at weeks 6, 12, 14 and 16; in NZB/W, weeks 6, 12, 18 and 28; in BXSB.*Yaa*, weeks 6, 12, 16 and 20; and in Tlr7.Tg6, weeks 6, 12, 16 and 28. These sampling weeks are referred to as pseudotimes 1,2, 3 and 4.

#### 2.1.3 Sex as a biological variable

The human dataset includes both males and females participants. The MRL^lpr/lpr^ and NZB/W F1 mice and their respective controls were females, while the BXSB.*Yaa* and Tlr7.Tg6 mice were males. In these spontaneous models, disease development is driven primarily by the underlying genetic alterations rather than by biological sex. These genetically driven mechanisms are distinct from sex−related immunological differences observed in human SLE. Therefore, the models used here should not be interpreted as reflecting sex−specific disease variation. Instead, their phenotypes arise from the engineered genetic modifications themselves.

#### 2.1.4 Mouse sampling of blood, kidney and spleen

At each time point, 10 animals from each SLE model and 5 controls were sacrificed (**Supplementary Table 2**). Mice were anesthetized by an intraperitoneal injection of 100 μg/g body weight ketamine and 16μg/g body weight xylazine. After the anesthesia, blood was collected through cardiac puncture and transferred into ethylenediaminetetraacetic acid (EDTA)-coated tubes (Vacutainer, Beckman Dickinson). The animals were perfused with cold phosphate-buffered saline (PBS), and the spleen and both kidneys were collected into cold PBS. Whole anticoagulated blood was centrifuged for 5 minutes at 500 g and 4°C to separate the plasma and stored at -80°C. The cell pellets were resuspended in a 10 ml red blood cell lysis buffer (RBCL; 155 mM NH4Cl, 12 mM NaHCO3, 0.1 mM EDTA) and incubated for 5 minutes at room temperature. Leukocyte pellets were washed with cold MACS buffer (Miltenyi) and resuspended in the same volume of MACS buffer as the initial blood volume. 200 ul of the cell suspension were centrifuged for 1 minute at 10,000 g, the cell pellet was immediately frozen on dry ice and stored at -80°C until RNA extraction. A fragment of the spleen was homogenized, and red blood cells were lysed as described above. Splenocytes were resuspended in MACS buffer, counted and analyzed by flow cytometry.

#### 2.1.5 Transcriptome profiling

RNA was extracted from snap-frozen white blood cell pellets, and homogenized spleen and kidney pieces using the RNeasy96 kit (Qiagen). For library synthesis, 400 ng of total RNA from the solid tissues and 100 ng of total RNA from white blood cells were used with the TruSeq Stranded mRNA HT kit (Illumina). The libraries were quantified using qPCR with the PerfeCTa NGS kit (Quantart Biosciences), and equimolar amounts of samples were pooled. Pooled samples were clustered on a high-output flow cell using the HiSeq SR Cluster kit v4 and the cBot instrument (Illumina). Subsequently, 50 cycles of single-read sequencing were performed on a HiSeq2500 instrument using a HiSeq SBS kit v4 (Illumina). On average, libraries were sequenced at 11.3 ± 2.95 million reads.

Reads were trimmed at the 3’ end for low quality (Q<20) and for adapter sequences using the cutadapt software (17). Reads shorter than 25 nucleotides were discarded. The resulting reads were aligned against the GRCm38 mouse assembly using the STAR program (18) with default parameters. Gene expression quantification was performed by the RSEM program (19) using the GENCODE annotation (20). Genes with at least 10 reads in at least 5 samples were retained for further analysis: 18,936 in spleen, 16,225 in blood and 18,491 in kidney.

#### 2.1.6 Flow cytometry profiling

Spleen samples were analyzed by flow cytometry using 3 multi-color panels. Panel 1 included B cells (B220+), and other subpopulations with specific markers CD93, B220, IgD, IgM CD21, CD23, CD5. Panel2 included T cells (CD5) and other populations defined by CD4, CD8, CD25, CD49b, CD44, CD62L. Panel 3 included plasma cells (PCs) (CD138), granulocytes (CD11b), neutrophils/eosinophils (CD11b, Gr-1), monocytes (CD11b, CD115), pDCs (CD11c, SiglecH) and cDCs (CD11c, SiglecH, CD8a, CD11b). The gating strategy and definition of all populations are provided in **Supplementary Figure 1**, and antibody commercial references are included in **Supplementary Table 3**. Samples were stained with the antibody cocktails in a FACSVerse (BXSB.*Yaa* and Tlr7.Tg6) and a FACSCanto II (NZB/W and MRL*^lpr/lpr^*). The two cytometers were aligned using compensation beads at the beginning of the study, and fluorescence signals were standardized using 8-peak beads, as described before (21). The compensation matrices and cell proportion quantification were calculated using the FlowJo.v10.7 program (22).

#### 2.1.7 Serology profiling

Serum cytokines and autoantibodies were quantified using the Luminex technology. The panels were designed to include cytokines and autoantibodies associated with different systemic autoimmune diseases in humans. Cytokines were analyzed using the Cytokine & Chemokine 36-plex Mouse ProcartaPlex Panel 1 Kit (Thermo Fisher). It included several interleukines (IL-10, IL-3, IL-1ß, IL-2, IL-4, IL-5, IL-6, IL-22, IL-9, IL12p70, IL-13, IL- 27, IL-23, IL-17A, IL-15/IL-15R, IL-1α, IL-28, IL-18, IL-31), interferons (INF-α, INF-Ɣ), monocyte-colony stimulating factor (M-CSF) granulocyte and monocyte colony stimulating factor (GM-CSF), granulocyte colony stimulating factor (G-CSF), and other cytokines such as leukemia inhibitory factor (LIF), interferon gamma-induced protein 10 (IP-10), growth-regulated oncogene-alpha (GRO-α, also known as CXCL-1), RANTES (regulated on activation, normal T cell expressed and secreted), tumor necrosis factor-alpha (TNF-α), macrophage inflammatory proteins (MIP-1α, MIP-1β, MIP-2), monocyte chemoattractant proteins (MCP-1, MCP-3), epithelial-derived neutrophil-activating peptide 78 (ENA-78), and eotaxin. The autoantibodies quantified included anti-histone, anti-Jo1, anti-centromere (anti-CENP-B), anti-double-stranded DNA (anti-dsDNA), anti-Sjögren’s-syndrome-related antigen A (anti-SSA/La), anti-Sjögren’s-syndrome-related antigen B (anti-SSB/Ro), anti-ribonucleoprotein (anti-RNP), and anti-Smith (anti-Sm) and were quantified by adapting the Athena ANA-II Plus Test System kit (Zeus Scientific), using a biotinylated anti-mouse-immunoglobulin G (IgG) antibody as a secondary detection antibody.

### 2.2 Statistical analysis

#### 2.2.1 Data values normalization

All molecular layers were transformed to normalize their distributions. Gene expression values were normalized using the varianceStabilizingTransformation function from the DESeq2 R package (23). Cell proportions, cytokines, and autoantibody values were log10(x+1) transformed.

#### 2.2.2 Transcriptional module annotation

Mouse gene-expression values were summarized using the GSVA R package (24) into a human immunological module set (25). The homologene database from NCBI (26) was utilized to find homologous genes between human and mouse. In total, 7,892 (69%) homologs were found between mouse and human out of the 11,465 genes annotated in the modules. Modules with less than 10 homologs were discarded.

#### 2.2.3 Longitudinal differential expression analysis

Linear regression analyses with an interaction term between group and pseudotime were conducted to identify differential longitudinal molecular entities. The model is represented as follows: Y = β_0_ + β_1_X_1_ + β_2_X_2_ + β_3_X_1_X_2_ + ϵ, where Y represents the molecular feature quantification, X_1_ represents the mouse groups and X_2_ represents the pseudotime values (1–4). Interaction term analysis identifies the association between molecular entities, mouse model changes and pseudotime. To exclude significant values driven by changes at a single time point, the linear relationship between molecular quantification and pseudotime, solely in the SLE model was conducted as: Y = β_0_ + β_1_X_2_ + ϵ. Overall, most molecular entities significant for the interaction term were also significantly associated with the pseudotime (FDR < 0.05): 294 out of 386 transcriptome modules, 7 out of 7 autoantibodies, 30 out of 31 cytokines, and 20 out of 21 cell type proportions. Significant entities for both linear models were selected for further analyses, indicating molecular differences that either increase or decrease over time.

#### 2.2.4 Time point cross-sectional analysis

Individual time point analyses were conducted by means of linear regression models (FDR < 0.05), change direction was confirmed with the longitudinal analysis. Longitudinally variable molecular features were classified as early or late based on the time-point analyses. Molecular features with significant differences in 3 or 4 time points were classified as early dysregulations, while late dysregulations were defined as molecular features with significant differences only at pseudo-times 3 and/or 4. Gene functionalities at each pseudo-time were assessed by Gene Set Enrichment Analysis (GSEA) implemented in clusterProfiler R package (27) using MgSigDB hallmark gene set (28).

#### 2.2.5 Molecular layer integration

Molecular integration was performed with MEFISTO (29), which based on factor decomposition of the multiple views, summarizes relationships that change gradually over time. MEFISTO was run using the MOFA2 framework with mainly default model, training and data options. The model was initialized with fifteen factors, to give the model enough latent dimensions to capture shared, view-specific, and covariate-associated variation, while letting the inference procedure down-weight unneeded factors. Data were not centered by group or additionally scaled beyond the normalization described above. Transcriptome data followed their recommended scaling procedure, while the other datasets were log-transformed to achieve a comparable scale. Further scaling or centering could distort biological effect sizes, amplify weak signals, or eliminate biologically relevant shifts between groups. Model training was performed using standard convergence settings without warping, as the sampling points had been selected to align with phenotype progression. SLE patients were classified into five categories based on their SLEDAI-2K: no activity (SLEDAI-2K=0), mild (1 to 5), moderate (6 to 10), high (11–19) and very high (>=20). These categories were based on previous publications (30, 31) and were assumed to be comparable in terms of severity of the disease with the pseudo-times selected for each mouse model (1 low, 2 moderate, 3 high and 4 very high). All autoantibodies, cytokines and cell populations were included in the analysis for both mouse and human. Transcriptional modules were preselected based on differential analysis between SLE patients and healthy controls (FDR < 0.05) including sex, age, sequencing batch and RNA integrity number as covariates (177 modules were selected).

## 3 Results

### 3.1 Mouse model sampling alignment with disease severity

**Figure 1:**
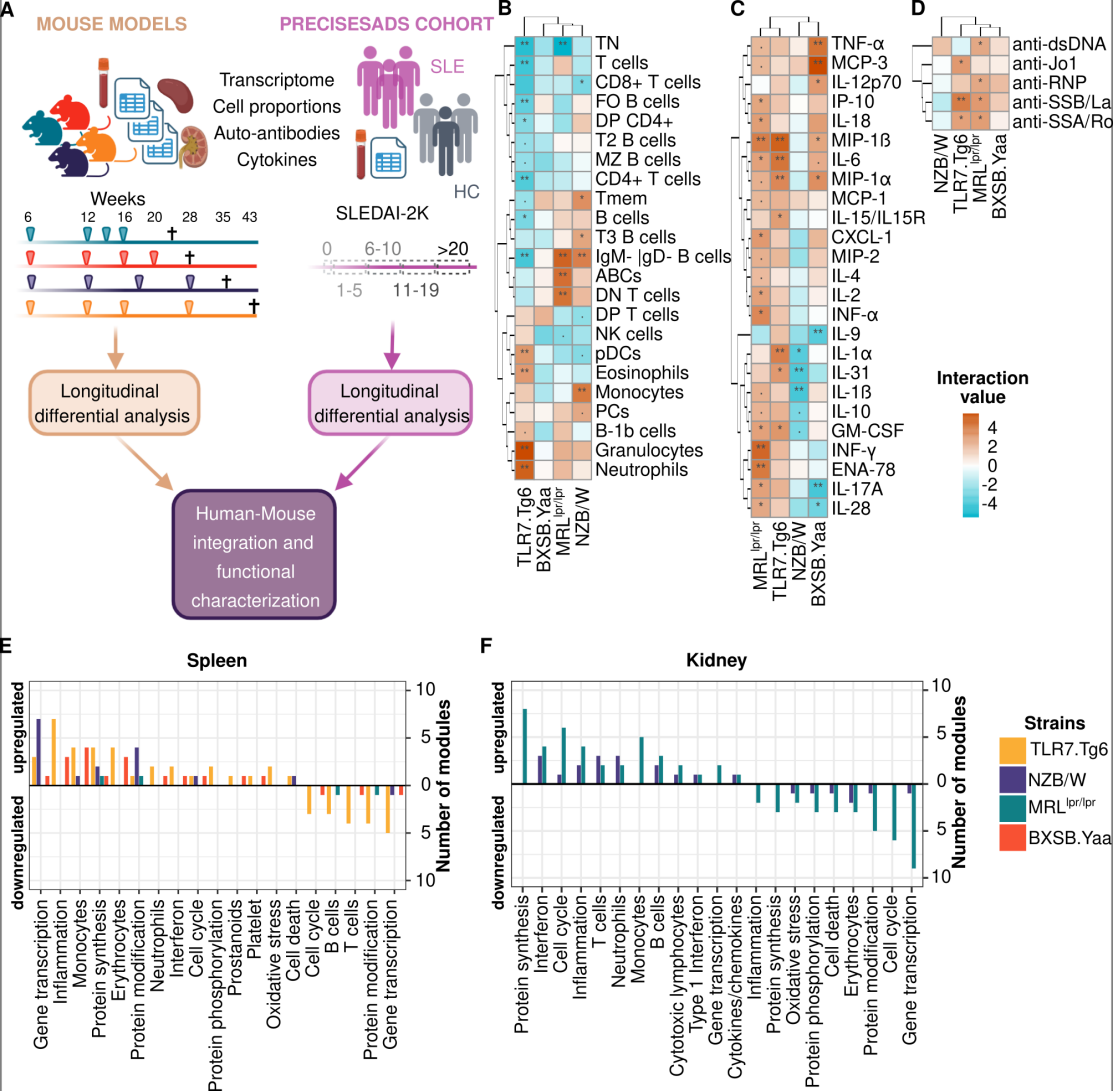
Molecular information shows differences between mouse models. **(A)** Analysis workflow: Different analyses were performed to investigate molecular differences between mice and humans. 50% mortality rates are represented as ✝. Longitudinal significant cell proportions **(B)**, cytokine levels **(C)**, and autoantibody levels **(D)** across mouse models are represented. Interaction term values with at least one comparison below 0.1 FDR are shown. Mouse models and features were grouped by hierarchical clustering. Interaction term significant values were indicated as follows:. FDR < 0.1, *FDR < 0.05, **FDR < 0.01. The number of longitudinal differential transcriptional immune modules is shown (interaction term FDR < 0.05) for the spleen **(E)** and the kidney **(F)** across the different mouse models. Significant modules were grouped by related molecular functions and sorted by the number and direction of change (from upregulation to downregulation in mouse models compared to their genetic controls). Mouse models are color-coded as follows: yellow (Tlr7.Tg6), purple (NZB/W), green (MRL*^lpr/lpr^*), and red (BXSB.*Yaa*). Modules without defined molecular functions (TBD) were excluded from the plot. Created with BioRender.com.

To investigate the molecular relationships between mouse models and humans, we profiled multiple molecular layers across different tissues and aligned the datasets as closely as possible between species. We selected four time points prior to the 50% mortality threshold in each model (**Figure 1A**) to capture early molecular changes associated with disease onset. These early time points aligned with the mild-to-moderate disease activity observed in the human SLE PRECISESADS cohort (average SLEDAI-2K: 5.94 ± 5.27).

In addition, the time points were selected to align disease severity across models, particularly regarding kidney involvement. In the selected time points, the MRL*^lpr/lpr^*, NZB/W and BXSB.*Yaa* models exhibited elevated urine albumin-to-creatinine ratios (ACR) at later time points, indicating kidney dysfunction (**Supplementary Figure 2A**). This trend paralleled the increase in serum creatinine levels and the presence of abnormal urine creatinine ratios (>0.2) observed in human patients grouped by SLEDAI-2K (**Supplementary Figures 2B, C**). The Tlr7.Tg6 model, known to exhibit the slowest disease progression among the four selected models, did not show abnormal ACR levels even at later time points. Nevertheless, histological analysis of kidney tissue at week 28 revealed evidence of tissue damage (Data not shown), despite the absence of noticeable changes in ACR.

### 3.2 Dysregulation of specific spleen cell populations characterizes each of the different mouse models

We applied linear regression models with interaction terms to assess how cell-type expansions related to phenotype progression over time (see Methods). In the Tlr7.Tg6 model, most lymphoid populations, including B and T cell subsets, were significantly depleted (**Figure 1B**). Naïve T cells (TN) showed the strongest depletion, a feature common to all models (**Figure 1B**) but occurring at different times (**Supplementary Figure 3A**). Both the MRL*^lpr/lpr^* and NZB/W models showed expansions of specific T cell subsets, including memory (Tmem) and double-negative T cells (DN T cells) (**Figure 1B**; **Supplementary Figure 3A**). In the B cell compartment, both the MRL*^lpr/lpr^* and NZB/W models showed significant overrepresentation of class-switched B cells (IgM- IgD- B cells in **Figure 1B**; **Supplementary Figure 3A**), whereas age-associated B cells (ABCs) were expanded specifically in the MRL*^lpr/lpr^* model (**Figure 1B**; **Supplementary Figure 3A**). Time point analyses revealed additional expansions: early expansion of plasma cells (PCs) and marginal zone B cells (MZ B cells) in the MRL*^lpr/lpr^* and NZB/W models, respectively, and late expansion of transitional T3 B cells (T3 B cells) in the NZB/W model (**Figure 1B**; **Supplementary Figure 3A**).

The Tlr7.Tg6 and NZB/W models showed the largest expansions in the myeloid compartment, although the specific lineages involved differed between them (**Figure 1B**). Eosinophils and plasmacytoid dendritic cells (pDCs) expanded exclusively in the Tlr7.Tg6 model. Notably, pDC expansion was not found in the closely akin BXSB.*Yaa* model (**Figure 1B**). Monocytes expanded in the NZB/W model, while neutrophils were expanded in the MRL*^lpr/lpr^* and Tlr7.Tg6 models (**Figure 1B**), indicating active inflammation via different pathways. Neutrophils and monocytes changes were not significant in Tlr7.Tg6 and NZB/W time point analyses, respectively, suggesting latter expansions compared to the MRL*^lpr/lpr^* model (**Supplementary Figure 3A**).

### 3.3 Serum cytokine levels related mostly with cell type expansion in the spleen and revealed an important contribution of macrophages in the BXSB.*Yaa* model

Serum cytokine levels were measured and the panel was selected based on human autoimmunity association. In order to identify cytokines changing over time, interaction terms within linear regression models were used as in the previous analysis.

Overall, the MRL*^lpr/lpr^* model exhibited the highest cytokine production, with significant upregulation across most analyzed cytokines. Particularly, the overexpression of TNF-α, IL-4, IL-2, and IL-17A (**Figure 1C**) which may be related with the DN T cells expansion (32, 33). This model also showed the highest increase in type I, II (IFN-α and IFN-γ) and III (IFN-λ, IL-28) interferons (**Figure 1C**). The timing of cytokine overproduction varied: IFN-γ and IP-10 increased early, IFN-α increased later, and IFN-λ appeared at the latest time points (**Supplementary Figure 3B**). Another prominent cytokine and chemokine group may involve neutrophil production and activation, marked by GM-CSF, ENA78, MIP-1α and MIP-1β overproduction. These last two cytokines are also known to induce the release of IL-1, IL-6, and TNF-α (34), all of them overproduced in the model (**Figure 1C**).

The Tlr7.Tg6 model also displayed marked cytokine overproduction, including GM-CSF, MIP-1α, MIP-1β, and IL-6 (**Figure 1C**), although these increases occurred later than in the MRL*^lpr/lpr^* model (**Supplementary Figure 3B**). The higher cytokine levels in the Tlr7.Tg6 model correlated with extensive neutrophil expansion as compared to MRL*^lpr/lpr^* (**Figure 1B**). The model also showed an eosinophil-specific expansion, potentially related with MIP-1α and MIP-1β overproduction, which are known to activate eosinophil and other myeloid populations (35). Additionally, IL-31 was also overproduced and potentially associated with eosinophil and dendritic cell activity (36, 37) (**Figure 1C**).

MCP-3 regulates macrophage function and monocyte recruitment (38), whereas IL-12 (IL-12p70) and TNF-α are key pro-inflammatory cytokines produced by macrophages in the affected tissues (39). All these were highly upregulated in the BXSB.*Yaa* serum, while IL-9, a known anti-inflammatory cytokine for macrophages (40), was downregulated (**Figure 1C**). These results suggest that macrophages play a central role in shaping the BXSB.*Yaa* phenotype. A shared neutrophil/macrophage-related cytokine core (MCP-3, MIP-1α, MIP-1β, IL-6, and TNF-α) was identified between MRL*^lpr/lpr^* and BXSB.*Yaa* models (**Figure 1C**). Interestingly, the BXSB.*Yaa* model showed earlier overproduction of MIP-1α, MIP-1β, and TNF-α, while MCP-3 and IL-6 appeared later, suggesting different inflammatory initiation pathways and should be further studied in relation to human SLE pathogenesis.

The NZB/W model displayed a different cytokine pattern compared with the other models. Key proinflammatory cytokines, such as IL-1α and IL-1β, are downregulated over time in the model (**Figure 1C**). This distinct cytokine profile suggests that the NZB/W model follows a different autoimmune trajectory, potentially influencing both its disease progression.

### 3.4 Autoantibody specificities and timings differentiate SLE mouse models

Autoantibodies associated with human autoimmunity were selected and measured in the serum. The MRL*^lpr/lpr^* and NZB/W models exhibited earlier overproduction of autoantibodies compared to the BXSB.*Yaa* and Tlr7.Tg6 models. The BXSB.*Yaa* model had the fewest autoantibody specificities (**Supplementary Figure 3C**), while the NZB/W model had the most, excluding anti-RNP antibodies (**Figure 1D**; **Supplementary Figure 3C**). However, longitudinal analysis failed to detect changes in the NZB/W model, likely due to the slower autoantibody production from its NZW genetic background, leading to smaller differences over time.

Classic SLE-associated anti-dsDNA autoantibodies increased in all models except Tlr7.Tg6 model (**Figure 1D**), occurring earlier in the MRL*^lpr/lpr^* and NZB/W models than in the BXSB.*Yaa* model. Anti-SSA/Ro and anti-SSB/La autoantibodies were highly upregulated in the Tlr7.Tg6 and MRL*^lpr/lpr^* models (**Figure 1D**), consistent with high interferon production (**Figure 1C**). However, these differences were undetectable in the time-point analysis of the MRL*^lpr/lpr^* model, likely attributable to its delayed overproduction of cytokines (**Supplementary Figure 3C**). The NZB/W model also showed overproduction of anti-SSA/Ro and anti-SSB/La antibodies, but was not associated with the interferon production (**Figure 1C**).

Anti-Smith (anti-Sm), anti-centromere B, and anti-histone autoantibodies were significantly upregulated early in the MRL*^lpr/lpr^* model (**Supplementary Figure 3C**), but were not significant in the longitudinal analysis (**Figure 1D**). Anti-Jo1 autoantibodies were overproduced in both the NZB/W and Tlr7.Tg6 models at different time points (**Supplementary Figure 3C**).

### 3.5 *Tlr7*-related models showed greater dysregulation of the spleen transcriptome dysregulation

The transcriptome analysis revealed molecular dysregulation in both the spleen and kidney across all mouse models. Notably, the spleen showed pronounced dysregulation in both the Tlr7.Tg6 and the BXSB.*Yaa* models (**Figure 1E**; **Supplementary Figure 4A**). In contrast, few or no differentially expressed modules were observed in blood (**Supplementary Figure 4B**), further supporting the spleen as the more informative tissue for studying immune dysregulation in these models (similar findings were observed at the individual gene level, Additional Files 1-4).

Transcriptional changes in the spleen aligned with the molecular dysregulations from other molecular layers. In the Tlr7.Tg6 model, interferon-related, inflammatory, and neutrophil-associated modules were upregulated, whereas lymphoid modules were downregulated (**Figure 1E**; **Supplementary Figure 4A**). Module dysregulation was consistent with the changes observed in spleen cell proportions and with the cytokine profiles (**Figures 1B, C**). The BXSB.*Yaa* model showed upregulation of monocyte and inflammatory modules in the spleen (**Figure 1E**; **Supplementary Figure 4A**). Although monocyte-related transcriptional modules did not coincide with an increased monocyte proportion, the cytokine profiles indicated a major role for macrophage activation (**Figure 1C**).

### 3.6 Interferon-related transcriptome dysregulation was primarily observed in the kidneys of MRL*^lpr/lpr^* and NZB/W mice

The kidney transcriptome in both MRL*^lpr/lpr^* and NZB/W models displayed a significant upregulation of interferon-related modules (**Figure 1F**, **Supplementary Figure 4C**). This interferon signature was reflected in serum cytokines in the MRL*^lpr/lpr^* model but not in the NZB/W mice (**Figure 1C**), indicating that in the latter, the signature was largely kidney-specific. Both models also showed upregulation of neutrophil and lymphoid-related modules, consistent with an increased immune-cell infiltration into the kidney (**Figure 1F**).

### 3.7 Correlation of blood and spleen transcriptomes validates blood as surrogate for spleen immune monitoring

**Figure 2:**
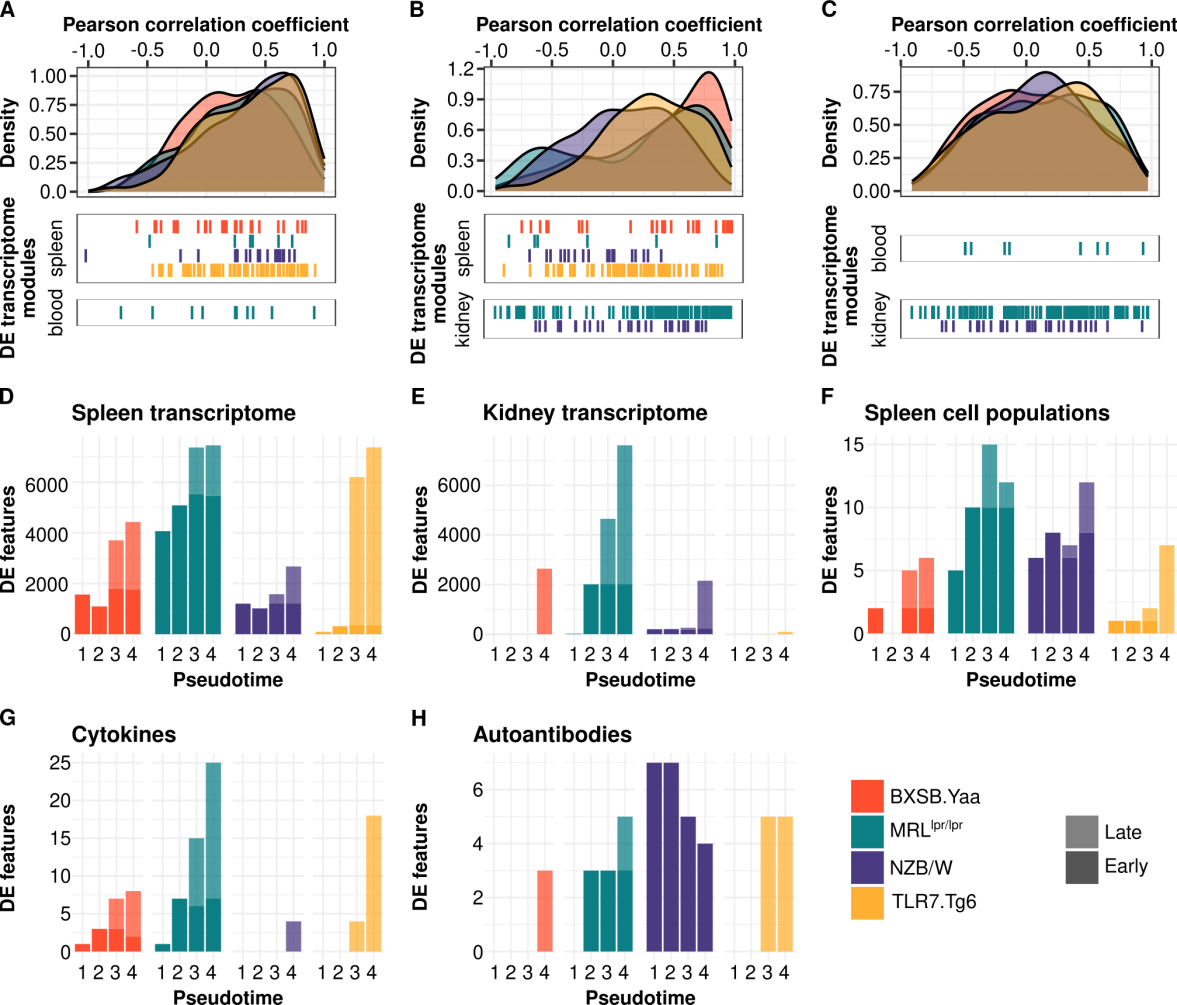
Transcriptome correlations and time point analyses reveal different dysregulation timings across molecular layers and tissues. Density plots for Pearson correlation coefficients are presented for all transcriptional modules by biological sample pairs: spleen-blood **(A)**, spleen-kidney **(B)**, and blood-kidney **(C)**. Significant differentially expressed modules (interaction term FDR < 0.05) are depicted per biological sample and mouse model below the density plots. Bar plots display the cross-sectional analysis at different time points for the spleen transcriptome **(D)**, kidney transcriptome **(E)**, spleen cell proportions **(F)**, cytokines **(G)**, and autoantibodies **(H)**. The differential features per each time point (FDR < 0.05) were classified as early or late. Mouse models are color-coded as follows: yellow (Tlr7.Tg6), purple (NZB/W), green (MRL*^lpr/lpr^*), and red (BXSB.*Yaa*).

To investigate the potential sharing of immunological signatures across different tissues, Pearson correlation coefficients between tissues were calculated for all the immunological transcriptional modules for each mouse model (**Figures 2A–C**). The analysis revealed clear correlations between spleen and blood across all models, with predominantly positive correlation values for the differentially expressed modules (**Figure 2A**; **Supplementary Figure 5A**). In contrast, correlations between spleen and kidney, or blood and kidney, were markedly weaker (**Figures 2B, C**). Despite these overall weaker correlations, certain molecular signatures showed strong cross-tissue correlations in specific models. For example, a myeloid-inflammatory signature, characterized by monocytes and neutrophils activity, showed significant correlations between spleen and kidney in the BXSB.*Yaa* and MRL*^lpr/lpr^* models (**Supplementary Figure 5B**). Similarly, an interferon signature was observed between blood and kidney in the Tlr7.Tg6 and MRL*^lpr/lpr^* models (**Supplementary Figure 5C**).

### 3.8 Timing of transcriptome and cytokine dysregulation associated with severity and disease progression

We next examined how molecular dysregulation related to disease severity and disease progression in the mouse models. Molecular entities were categorized as early or late based on the initial time point when dysregulation was observed and its continuity over time (see Methods).

Across all models, transcriptome dysregulation appeared first in the spleen and later in the kidney (**Figures 2D, E**). These changes were earlier and stronger in the MRL*^lpr/lpr^* and BXSB.*Yaa* models compared to NZB/W and Tlr7.Tg6 models. Splenic dysregulation was associated with changes in cell proportions (**Figure 2F**) and synchronized with cytokine dysregulation (**Figure 2G**). These findings suggest that early immune changes in the spleen may drive subsequent alterations in target organs, potentially including the cell infiltration observed in the kidney. Furthermore, the early molecular changes in the spleen compared to the kidney may in part explain the lower correlations of immune signatures between these organs (**Figure 2B**).

Functional pathway enrichment analysis from the first time point revealed dysregulation of immune gene sets in the spleen of all models (**Supplementary Figure 6A**). However, kidney dysregulation in less aggressive NZB/W and Tlr7.Tg6 models, was less significant and/or appeared only at the latest time points (**Supplementary Figure 6B**). Similar patterns were observed for other immune-related gene sets, such as IL2-STAT5 signaling, TNF-α signaling via NFKB, and KRAS signaling (**Supplementary Figures 6A, B**). In the same line, the apoptosis gene set, indicating initiation of tissue damage, was dysregulated earlier in the most aggressive MRL*^lpr/lpr^* and BXSB.*Yaa* models (**Supplementary Figure 6B**). This highlights the association between phenotype timing and the transcriptome dysregulation in the different tissues.

Autoantibody overproduction either preceded or coincided with the emergence of kidney transcriptome dysregulation (**Figures 2E, H**), suggesting a temporal relationship between autoantibody development and molecular changes in the kidney.

### 3.9 Human SLE molecular signatures were shared between patients and specific mouse models

The mouse-centered analyses revealed molecular signatures that may drive or initiate disease phenotypes. To investigate whether similar molecular dysregulations underlie the phenotypes in mouse models and in humans, we conducted a disease severity-oriented integration using factor analysis (29).

In mouse models, disease severity progresses in a largely monotonic manner, enabling the use of pseudotime as a reliable proxy for identifying molecular dysregulation. In humans, however, SLE follows a non−linear course marked by flares and remissions, rendering time since diagnosis an inappropriate indicator of disease severity. In fact, no significant associations were observed between disease duration and molecular features (Additional file 5). The SLEDAI-2K is the most used disease activity index in clinical practice, and it has been previously used to correlate molecular information with successful results (5, 6). Indeed, SLEDAI-2K values were significantly associated with well-known molecular features in our SLE cohort (**Supplementary Figure 7**). Therefore, for integration we defined disease severity in the mouse models as pseudotimes and categorized SLEDAI-2K groups in humans (see Methods).

**Figure 3:**
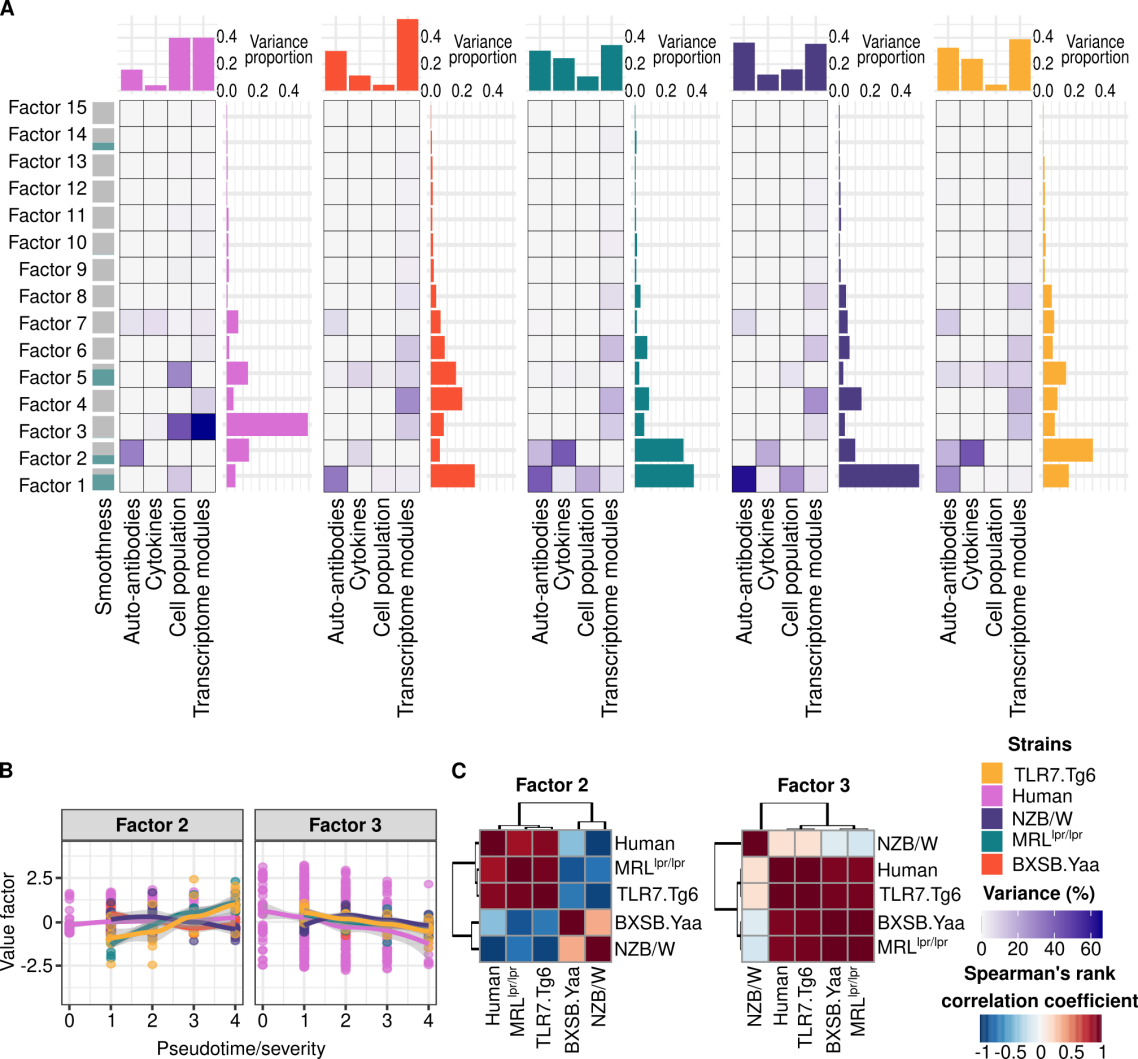
Longitudinal molecular factors are comparable between mouse models and SLE patients. **(A)** Explained variance proportion is shown per factor (rows), molecular layer of information (columns), human and mouse strain (panels). Factors’ longitudinal trends are represented on the left side of the representation in terms of smoothness. **(B)** Trend along severity is shown for each significant factor per group. Factor values per severity and group are summarized by means of loess regression. Trend along severity is shown for each significant factor per group. Factor values per severity and group are summarized by means of loess regression. **(C)** Factor value correlations between human and mouse models are depicted for the significant factors. Mouse models are color-coded as follows: yellow (Tlr7.Tg6), purple (NZB/W), green (MRL*^lpr/lpr^*), and red (BXSB.*Yaa*). Human results are colored pink.

The integration yielded fifteen factors, of which four (1, 2, 3, and 5) met the criteria for smoothness (dependency on severity) and explained variance (**Figure 3A**). These four factors were independent (**Supplementary Figure 8A**) and accounted for 68.07 ± 9.85% of the overall observed variance. All molecular layers were represented in the factors, but humans had a diminished cytokine-related signal due to the limited number of cytokines profiled (**Figure 3A**). Factors 2 and 3 showed consistent severity−related trends in both humans and mouse models (**Figure 3B**, linear regression p-value < 0.05), while factors 1 and 5 were mouse-specific (**Supplementary Figure 8B**).

Factor 1 increased over time in all mouse models (**Supplementary Figure 8C**), driven by the expansion of activated B cells (ABCs and IgM- IgD- B cells), memory T cells, neutrophils, and the depletion of naïve T cells (**Supplementary Figure 9A**). Cell proportion profiles in the spleen primarily originate from the MRL*^lpr/lpr^* and NZB/W mouse models (**Figure 3A**), while the presence of anti-dsDNA, anti-RNP, and anti-histone autoantibodies was consistent across all mouse models within this factor (**Supplementary Figure 9B**). Factor 5 showed positive trends in the MRL*^lpr/lpr^*, BXSB.*Yaa*, and Tlr7.Tg6 models (**Supplementary Figure 8B**), distinguishing them from the NZB/W model and from humans (**Supplementary Figure 8C**). Cell populations and cytokines played major roles in this factor. Specifically, CD4+ T cells and CD4+/CD8+ double positive T cells (DP T cells) drove the factor in the Tlr7.Tg6 model (**Figure 3A**; **Supplementary Figure 9A**), whereas myeloid-related cytokines (MCP-2, MIP-1α, IL-6, and TNF-α) predominantly influenced the factor in the BXSB.*Yaa* model (**Supplementary Figure 9C**).

**Figure 4:**
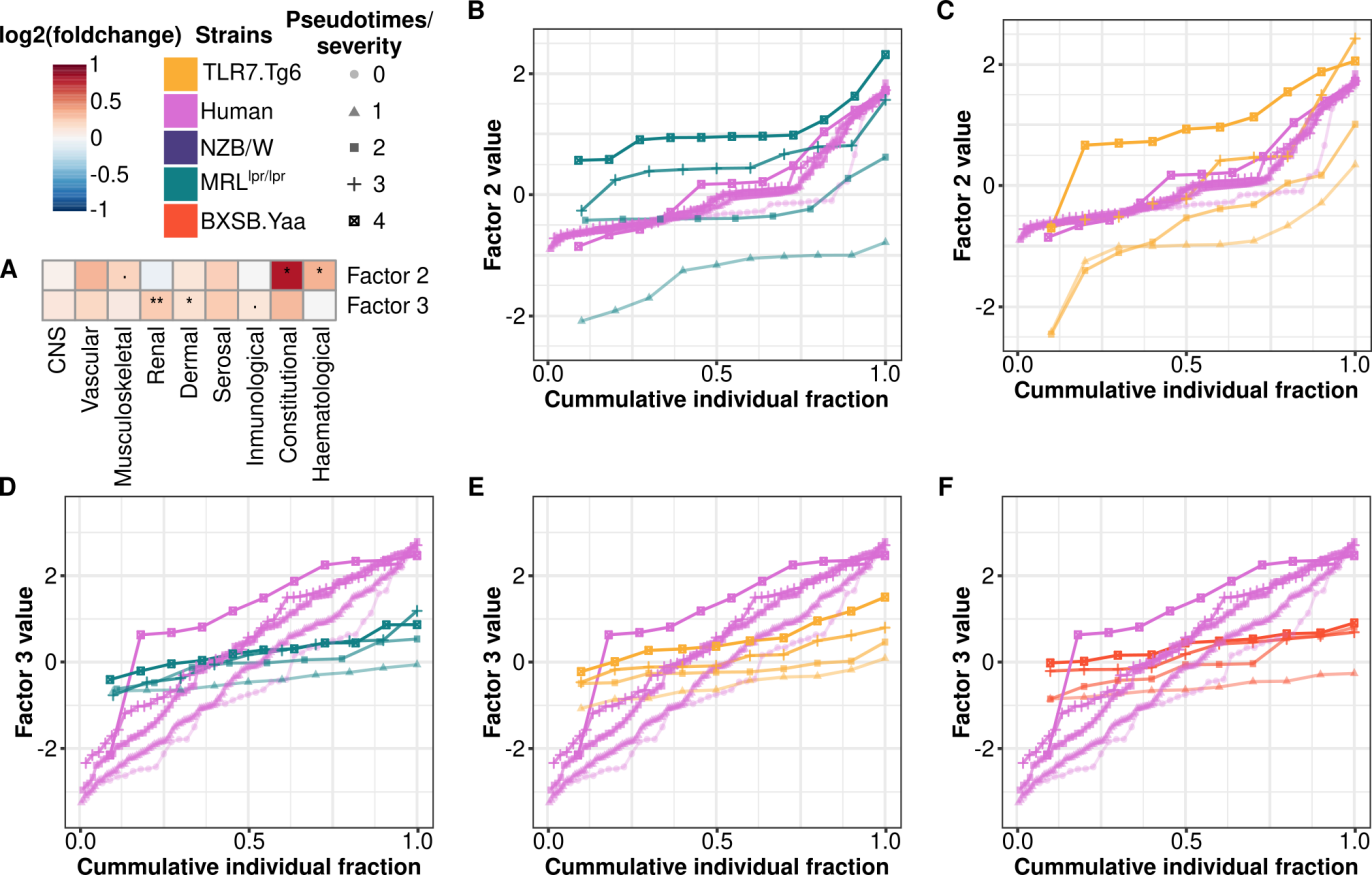
Pathogenic factors associate with specific clinical domains and time points in the different mouse models. **(A)** log_2_(fold change) between significant factor values and SLEDAI clinical domains values are shown (Wilcoxon signed rank test:.p-value < 0.1, *p-value < 0.05, **p-value < 0.01). The cumulative distributions per severity/pseudotime of individual fractions based on the factor values are depicted by mouse model and compared with human: **(B)** Factor 2 for MRL*^lpr/lpr^*, **(C)** Factor 2 for Tlr7.Tg6, **(D)** Factor 3 for, **(E)** Factor 3 for Tlr7.Tg6 and **(F)** Factor 3 for BXSB.*Yaa*. Mouse models are color-coded as follows: yellow (Tlr7.Tg6), purple (NZB/W), green (MRL*^lpr/lpr^*), and red (BXSB.*Yaa*). Human results are colored pink.

Factors 2 and 3 were shared between humans and mice. Factor 2 was primarily influenced by autoantibodies against extractable nuclear antigens, particularly anti-SSA/Ro (**Supplementary Figure 9B**), which drove the positive trend correlations between humans, the MRL*^lpr/lpr^* and the Tlr7.Tg6 mouse models (**Figures 3B, C**). Notably, these two mouse models contributed significantly to the explained variance (**Figure 3A**) through the overexpression of cytokines measured in both species, such as IP-10, MIP-1β, IL-6, IL-17A and IL-22 (**Supplementary Figure 9C**). These cytokines have previously been associated with the regulation of interferon production, as well as other cytokines contributing to this factor that were only profiled in mice (**Supplementary Figure 9C**), including IFN-γ and IFN-λ (IL-28). Based on the SLEDAI-2K decomposition into its clinical domains, it was found that this interferon-driven factor 2 was associated with constitutional and hematological clinical domains (**Figure 4A**). Factor 3 showed significant downward trends across models, except NZB/W (**Figure 3B**), mirroring the pattern observed in humans (**Figure 3C**). Factor 3 was the factor explaining the most variance in humans (**Figure 3A**), mainly driven by neutrophil expansion (**Supplementary Figure 9A**) and myeloid inflammatory and leukocyte activation-related transcriptional module upregulation (**Supplementary Figure 9D**). The cytokines contributing to the factor included MMP-8, TGFB1, IL-1Ra (**Supplementary Figure 9C**), which are known to be released by neutrophils upon stimulation, and BLC (*CXCL13* gene), which has been associated with nephritis in both humans and mice (41, 42). In this line, the factor showed a strong association with the renal and cutaneous SLEDAI-2K domains (**Figure 4A**).

To identify the time points in each mouse model that most closely resemble the highest human factor levels, we plotted the cumulative distributions of factor values (**Figures 4B–F**). For the interferon-related factor, MRL*^lpr/lpr^* pseudotimes 2-3 (weeks 12-14) and Tlr7.Tg6 pseudotime 3 (week 16) closely resembled the distribution observed in severe SLE patients (**Figures 4B, C**). For the neutrophil-associated factor, none of the models reached the highest levels observed in severe human SLE, indicating the need for later time points to fully capture the molecular dysregulation of this pathway in humans (**Figures 4D–F**).

## 4 Discussion

Animal-based experimental setups are challenging due to heterogeneity, cost, time and ethical concerns. Moreover, experimental outcomes may be unsatisfactory if models fail to molecularly mimic the intended disease. This likely influenced the FDA’s decision to allow human drug trials without prior preclinical animal testing. Nonetheless, animal models have been useful in understanding immune regulation, molecular disease mechanisms (43) and assessing the efficacy of drug candidates prior to human clinical trials (10, 11). For complex disorders such as SLE, the molecular dysregulations driving phenotypic similarities in animal models remain incompletely understood. Therefore, a deeper understanding of these mechanisms in comparison with human disease is crucial for an efficient use of animal models. In this study, we longitudinally profiled multiple molecular layers in four spontaneous SLE mouse models and integrated these data with human molecular profiles. However, mouse model phenotypes and human disease do not progress in the same way. In mouse models, disease progression is largely monotonic, with worsening symptoms and molecular dysregulation increasing over time. In contrast, human SLE follows a non−linear course characterized by flares and remissions, making time since diagnosis an unsuitable metric for integration. Indeed, in our analyses most mouse molecular features exhibited monotonic changes over time, whereas no significant molecular dysregulation was associated with time since diagnosis in humans. Therefore, in this study we used pseudotime in mouse models and disease activity (SLEDAI−2K) in humans as comparable proxies of disease severity to guide the integration analysis. Although these measures are not biologically equivalent, they represent the most informative and context−appropriate indicators of disease burden within each system.

Our results showed that both disease severity and 50% mortality rates were associated with the magnitude and timing of molecular dysregulation, even when sampling was aligned according to model-specific disease severity. The MRL*^lpr/lpr^* model, characterized by its aggressiveness with 50% mortality rate at 5 months in females (44), showed the highest number of differentially expressed molecular entities and early dysregulations, likely contributing to its rapid and severe phenotype. In contrast, the BXSB.*Yaa* and NZB/W models, with milder phenotypes, displayed fewer differentially expressed genes in the spleen and delayed kidney dysregulation, consistent with their 50% mortality rates between 5 and 8 months (44, 45). The least aggressive Tlr7.Tg6 model, with 50% mortality exceeding 10 months (15), had the fewest differences, detectable only at the latest time point in the kidney. This supports the notion that disease severity is correlated with the extent and timing of molecular dysregulations. Moreover, specific temporal relationships between molecular layers and tissues were identified. Our findings indicated a sequential pattern in which molecular dysregulation first emerges in the spleen, followed by autoantibody production, and subsequently affects the kidney. This sequential escalation of events is mirrored at the functional level, where, for example, dysregulated immune pathways precede apoptosis triggering in the tissues.

While spleen is often used in mouse immunological studies, whole blood is utilized in human research as a non-invasive proxy for immune activity. Our findings showed good transcriptional correlations between blood and spleen in mice, suggesting that blood is a reliable proxy for the spleen. However, neither blood nor spleen fully captured the kidney’s molecular signature, as not all circulating immune cell types infiltrate tissues during the autoimmune process. Thus, it is reasonable to expect that not all immunological signatures observed in the spleen or blood would be present in the kidney.

**Figure 5:**
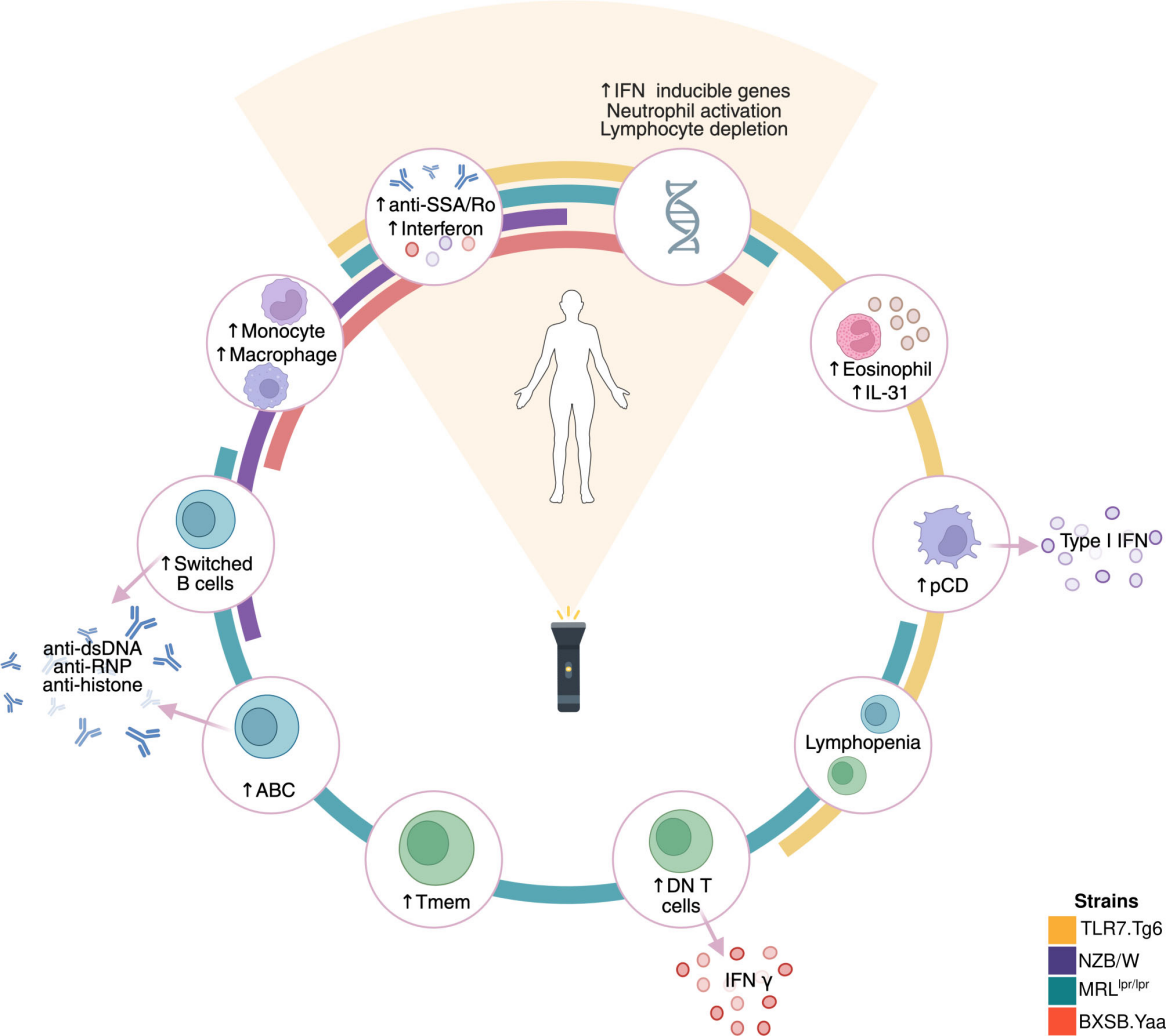
Molecular commonalities and specificities between SLE patients and spontaneous mouse models. Summary of the molecular signatures associated with the development of phenotypes in mouse models and the severity in human SLE. Molecular features associated with human SLE are depicted at 12 o’clock position in the plot. Each colored circular line represents a different mouse model: yellow (Tlr7.Tg6), purple (NZB/W), green (MRL*^lpr/lpr^*), and red (BXSB.*Yaa*). The surrounding molecular dysregulations were found to be associated with the development of the phenotype in at least one mouse model, defined by the circular lines. Created with BioRender.com.

Mouse model analyses uncovered diverse molecular dysregulations contributing to phenotype development, which showed continuous trends over time. These molecular dysregulations were consolidated within factor 1 during the integration analysis. This factor primarily involved immune cell migration and activation across different cell compartments. The B cell compartment showed an activation process, including an expansion of switched IgM-IgD-B cells (mainly in MRL*^lpr/lpr^* and NZB/W models) and ABCs (exclusive to MRL*^lpr/lpr^*) in the spleen. These cell types may be responsible for the overproduction of anti-dsDNA, anti-RNP, and anti-histone autoantibodies associated with this factor. Given the emerging significance of ABCs in SLE, our findings suggest that the MRL*^lpr/lpr^* model is particularly well suited for studying their role in SLE pathogenesis (46, 47). In addition, CD4+ T cell lymphopenia was identified, mirroring the characteristic reduction in CD4+ T cell compartments observed in SLE patients (48), marked by a decrease in naïve T cell populations and an increase in memory T cells. This process was particularly noted in the MRL*^lpr/lpr^* model and in the Tlr7.Tg6 at later time points. However, these molecular processes identified in mice were not recapitulated in humans (see **Figure 5** for a graphical overview), leaving their relevance in human disease unclear. Nevertheless, one of the factors shared between mice and humans (factor 3) included the downregulation in CD4+ and CD8+ T cell compartments, which might be associated with a reduction in their naïve compartments and related with the lymphopenia.

Whole-blood human transcriptome analyses have consistently revealed dysregulations in various molecular signatures associated with SLE. These include upregulation of interferon-inducible genes, activation of neutrophils, and lymphocyte depletion in both T and B cell compartments (5, 49–51). The cross-species integration revealed, for the first time, a core of shared molecular features representing these well-known SLE-associated signatures between species (**Figure 5**). The interferon signature stands out as one of the most studied immune pathways linked to human SLE. Factor 2 cytokine profiling, together with the established link between interferon production and anti-SSA/Ro autoantibodies (52, 53), and the absence of contributions from cell-type proportions, supports our inference that factor 2 reflects an interferon-inducible signature shared between humans and mice. Moreover, its minimal variation across human severities is consistent with prior evidence that the interferon signature persists even after disease remission (51). Based on our results, both the MRL*^lpr/lpr^* and Tlr7.Tg6 models are suitable for studying phenotypes driven by this interferon signature. The time points selected in our study are well suited to capture both early and late molecular changes underlying this interferon signature. Furthermore, our study unveiled an interferon dysregulation cascade in the MRL*^lpr/lpr^* model, initiated by the overproduction of IFN-γ possibly stemming from the expansion of DN T cells in the model, which have been linked to kidney damage in SLE (32). Conversely, in the Tlr7.Tg6 model, interferon production likely arises from expanded pDCs, known as major producers of type I interferons, and previously associated with disease activity in cutaneous lupus (53). These findings suggest distinct initiation pathways for the interferon signature in these mouse models, each associated with different SLE phenotypes. However, further experiments are needed to elucidate whether both pathways coexist or trigger the interferon signature in different subgroups of SLE patients.

Another significant peripheral blood molecular signature commonly associated with SLE patients involves neutrophil activation and/or expansion, often linked to the proliferation of low-density granulocytes (54). This signature has been consistently linked to lupus nephritis (5–7), underscoring its pathological relevance in the disease, and evidenced here by the factor-association with the renal SLEDAI-2K domain. Factor 3 captured this molecular pathway through a pronounced expansion of neutrophils coupled with an overexpression in neutrophil-related cytokines and transcriptional modules. Additionally, another well-established SLE-related signature, marked by a general depletion of B and T lymphocytes, was also encompassed within this factor and reflected in the transcriptome. Unfortunately, although the factor was present in the BXSB.*Yaa*, Tlr7.Tg6, and MRL*^lpr/lpr^* models, none of them exhibited values reaching the highest levels observed in humans. Among the profiled time points, the sharpest upward trend was observed in the Tlr7.Tg6 model, suggesting that it may be the most suitable model for profiling at longer time points, particularly given its lower aggressiveness. Notably, recent findings have demonstrated extensive sharing of neutrophil transcriptional programs between mice and humans (55), further underpinning these results.

Although main human SLE-related molecular signatures were captured by the factorization strategy, certain molecular signatures identified during regression analyses were not explained by any factor. For instance, DN T cells were dysregulated early in the MRL*^lpr/lpr^* model but did not align with any factor. This early expansion, and that of memory T cells, likely reflects the impaired apoptosis via the Fas-FasL pathway described in the model (56), allowing escape from activation-induced cell death. Both processes are linked to SLE pathogenesis (32, 57), yet were not captured by our strategy, likely because their sustained, pronounced expansion lacked a longitudinal trend. A similar scenario was observed for pDCs in the Tlr7.Tg6 model. In this case, pDCs expansion and overactivation are linked to the upregulation of the *Tlr7* gene (58). However, pDCs were not associated with any factor, as their expansion occurred at the latest time point of the model. Notably, this model showed an expansion of eosinophils and the overexpression of IL-31 and interferon-related proteins, which have been linked to inflammatory skin diseases (59, 60). Thus, the Tlr7.Tg6 model may be a promising candidate for studying cutaneous manifestations of lupus. In the NZB/W and BXSB.*Yaa* models, monocyte/macrophage-related signatures may contribute to phenotype development. In the NZB/W model, the signature was detected as an increased proportion of monocytes in the spleen, whether in BXSB.*Yaa* was shown based on cytokine and transcriptomic signatures. However, no factor captured these associations or their sharedness with human molecular signatures. Notably, although monocytosis is a characteristic feature of the BXSB.*Yaa* model, it has not been associated with disease severity in the model, unlike in the NZB/W model (61). In addition, the NZB/W model was the least represented in the factorization analysis. Its genetic control strain (NZW), while clinically healthy for much of its lifespan, is known to progressively develop autoantibodies, immunological abnormalities, and even nephritis (62). This gradual emergence of immune-related features in the control strain, which was also evident in our analyses, likely disrupts the monotonic trend required for our severity-based factorization approach. Overall, the lack of representation of these signatures in the factorization analysis likely reflects their temporal dynamics, which do not follow gradual severity-linked trajectories. Some signatures are dysregulated from the earliest time points and remain sustained, whereas others emerge only at the latest time point. In the case of NZB/W, the progressive increase of SLE-related features in the genetic control further complicates longitudinal association. Such non−monotonic patterns are inherently difficult to capture with a framework designed to identify longitudinal trends, representing an important limitation of this integration strategy.

A further limitation of the integration analysis stems from the imbalance in cytokine profiling between species, with a substantially broader cytokine panel available in mice compared to humans. This disparity likely reduced the contribution of cytokine signals to human factors and may have attenuated the detection of shared cytokine−driven pathways across species. Nevertheless, the main cytokine−representative factor (Factor 2) was consistently captured across all mouse models and shared with humans, largely due to the strong relationship between cytokine production and the autoantibody profiles observed. However, future studies incorporating more comprehensive and longitudinal cytokine or proteomic profiling in human cohorts, combined with single−cell approaches to increase granularity, may further enhance cross−species resolution and refine the identification of shared pathogenic pathways.

## 5 Conclusions

Among the four mouse models analyzed, Tlr7.Tg6 and MRL*^lpr/lpr^* most closely mirror the interferon and neutrophil-related molecular dysregulations observed in human SLE. These models exhibit distinct molecular characteristics during disease progression and varying degrees of severity, thereby offering flexibility depending on the specific experimental objectives or pathways under investigation. While the BXSB.*Yaa* and NZB/W models also demonstrated interesting signatures, such as monocyte/macrophage migration and activation or early and pronounced autoantibody production, none directly mirrored the molecular dysregulations observed in human SLE, at least based on our results. For the first time, this study directly integrates molecular information from SLE mouse models within the context of human SLE, shedding light on which mouse models and time points most faithfully recapitulate some specific molecular signatures associated with the human disease. We anticipate that these results will help to delineate future SLE mouse model-based experimental designs, thereby enhancing the quality and relevance of their outcomes.

## PRECISESADS clinical consortium

Lorenzo Beretta, Barbara Vigone, Jacques-Olivier Pers, Alain Saraux, Valérie Devauchelle-Pensec, Divi Cornec, Sandrine Jousse-Joulin, Bernard Lauwerys, Julie Ducreux, Anne-Lise Maudoux, Carlos Vasconcelos, Ana Tavares, Esmeralda Neves, Raquel Faria, Mariana Brandão, Ana Campar, António Marinho, Fátima Farinha, Isabel Almeida, Miguel Angel Gonzalez-Gay, Ricardo Blanco Alonso, Alfonso Corrales Martínez, Ricard Cervera, Ignasi Rodríguez-Pintó, Gerard Espinosa, Rik Lories, Ellen De Langhe, Nicolas Hunzelmann, Doreen Belz, Torsten Witte, Niklas Baerlecken, Georg Stummvoll, Michael Zauner, Michaela Lehner, Eduardo Collantes, Rafaela Ortega-Castro, M^a^ Angeles Aguirre-Zamorano, Alejandro Escudero-Contreras, M^a^ Carmen Castro-Villegas, Yolanda Jiménez Gómez, Norberto Ortego, María Concepción Fernández Roldán, Enrique Raya, Inmaculada Jiménez Moleón, Enrique de Ramon, Isabel Díaz Quintero, Pier Luigi Meroni, Maria Gerosa, Tommaso Schioppo, Carolina Artusi, Carlo Chizzolini, Aleksandra Dufour, Donatienne Wynar, Laszló Kovács, Attila Balog, Magdolna Deák, Márta Bocskai, Sonja Dulic, Gabriella Kádár, Falk Hiepe, Velia Gerl, Silvia Thiel, Manuel Rodriguez Maresca, Antonio López-Berrio, Rocío Aguilar-Quesada, Héctor Navarro-Linares, Yiannis Ioannou, Chris Chamberlain, Jacqueline Marovac.

## PRECISESADS flow cytometry consortium

Christophe Jamin, Concepción Marañón, Lucas Le Lann, Quentin Simon, Bénédicte Rouvière, Nieves Varela, Brian Muchmore, Aleksandra Dufour, Montserrat Alvarez,Carlo Chizzolini, Jonathan Cremer, Ellen De Langhe, Nuria Barbarroja, Chary Lopez-Pedrera, Velia Gerl, Laleh Khodadadi, Qingyu Cheng, Anne Buttgereit, Zuzanna Makowska, Aurélie De Groof, Julie Ducreux, Elena Trombetta, Tianlu Li, Damiana Alvarez-Errico, Torsten Witte, Katja Kniesch, Nancy Azevedo, Esmeralda Neves, Sambasiva Rao, Pierre-Emmanuel Jouve, Jacques-Olivier Pers.

## Data Availability

The datasets presented in this article are not readily available because of ethical and legal limitations. The PRECISESADS dataset allows the use of patient data exclusively for scientific research in autoimmune diseases and for non-profit purposes only. Access can be obtained by contacting the data stewardship team of ELIXIR Luxembourg at lcsb-datastewards@uni.lu.

## Supplementary material

Additional File 1 (xlsx): Statistical analyses for MRL*^lpr/lpr^*. Statistics from regression and correlation analyses are included in the file.

Additional File 2 (xlsx): Statistical analyses for BXSB.*Yaa*. Statistics from regression and correlation analyses are included in the file.

Additional File 3 (xlsx): Statistical analyses for NZB/W. Statistics from regression and correlation analyses are included in the file.

Additional File 4 (xlsx): Statistical analyses for Tlr7.Tg6. Statistics from regression and correlation analyses are included in the file.

Additional File 5 (xlsx): Statistical analyses for human samples. Statistics from regression and correlation analyses are included in the file.

## Glossary

ABCs: Age-associated B cells
ACR: Albumin-to-creatinine ratio
ANAs: Antinuclear antibodies
anti-CENP-B: Anti-centromere protein B
anti-dsDNA: Anti-double-stranded DNA
anti-RNP: Anti-ribonucleoprotein
anti-Sm: Anti-Smith antigen
anti-SSA/La: Anti-Sjögren’s-syndrome-related antigen A
anti-SSB/Ro: Anti-Sjögren’s-syndrome-related antigen B
CXCL-1: Chemokine (C-X-C motif) ligand 1
DN T cells: Double-negative T cells
DP T cells: Double-positive T cells
EDTA: Ethylenediaminetetraacetic acid
ENA-78: Epithelial-derived neutrophil-activating peptide 78
FDA: Food and Drug Administration
FDR: False discovery rate
G-CSF: Granulocyte colony-stimulating factor
GM-CSF: Granulocyte-macrophage colony-stimulating factor
GRO-⍺: Growth-related oncogene-alpha
GSEA: Gene Set Enrichment Analysis
IFN-⍺: Interferon-alpha
IFN-Ɣ: Interferon-gamma
IgG: Immunoglobulin G
IgM- IgD- B cells: Class-switched B cells
IL: Interleukin
IP-10: Interferon gamma-induced protein 10
MCP-1: Monocyte chemoattractant protein-1
MCP-3: Monocyte chemoattractant protein-3
M-CSF: Monocyte-colony stimulating factor
MIP-1⍺: Macrophage inflammatory protein-1 alpha
MIP-1ß: Macrophage inflammatory protein-1 beta
MIP-2: Macrophage inflammatory protein-2
MRL: Murphy Roths Large
MZ B cells: Marginal zone B cells
NZB: New Zealand Black
NZW: New Zealand White
PBS: Phosphate-buffered saline
PCs: Plasma cells
pDCs: Plasmacytoid dendritic cells
RANTES: Regulated upon activation, normal T cell expressed and secreted
SLE: Systemic lupus erythematosus
T3 B cells: Transitional T3 B cells
Tmem: Memory T cells
TN: Naïve T cells
TNF-⍺: Tumor necrosis factor-alpha
